# Impact of cardiorespiratory monitoring on the initiation of cardiopulmonary resuscitation in children. friend or foe?

**DOI:** 10.1186/2197-425X-3-S1-A747

**Published:** 2015-10-01

**Authors:** E Hoerner, K Schebesta, M Hüpfl, O Kimberger, B Roessler, Medical Simulation and Emergency Management Research Group, Department of Anaesthesia, Critical Care and Pain Medicine, Medical University of Vienna, Austria

**Affiliations:** General Anaesthesia and Intensive Care Medicine, Medical University of Vienna, Vienna, Austria

## Introduction

Survival rates from paediatric in-hospital cardiac arrests (CA) have not changed substantially in the last 30 years and range from 14 to 36%.[1] Recent studies emphasise that the early initiation of basic life support (BLS) within the first minutes is essential for survival and contributes to a better outcome.[2] Despite the importance of the first responders' interventions, it has been shown that their actions are often inadequate.[1] Little is known about how the presence of monitoring may influence life support measures. The aim of this study was to compare the times to initiation of first chest compressions in monitored versus non-monitored simulated paediatric CA scenarios.

## Objectives

Time to first chest compression was the primary outcome parameter. Secondary outcomes were type and frequency of resuscitation errors and subjective evaluation of the scenarios.

## Methods

This was a randomised, controlled, prospective trial. Sixty residents were randomized to either the monitoring or non- monitoring group. In both groups, the case history consisted of a six-month-old boy in CA. In the monitoring group, the simulator was connected to a monitor showing pulseless electric activity (sinus rhythm at a rate of 100 beats per minute but no peripheral oxygen saturation or blood pressure measurable), in the non-monitoring group there was no monitoring attached.

## Results

The time to first chest compression was significantly longer in the monitoring than in the non-monitoring group (135 ± 90 vs. 71 ± 26 seconds, p = 0.01). In this group, six participants (20%) did not initiate chest compressions within five minutes, compared to none in the non-monitoring group. Adherence to the current guidelines was also better in the non-monitoring group (p = 0.02). The participants' mean ratings of their overall performance were better in the non-monitoring than in the monitoring group (p = 0.03).

## Conclusion

Early and high-quality BLS is the key to optimal outcome after CA. This study indicates that monitoring can be a confounding factor delaying the start or even preventing BLS measures in the simulated paediatric cardiac arrest. This might be improved by specifically addressing this issue during training sessions.
